# Engineering smallholder common bean cropping systems with flowering plants increases hoverfly populations and crop yields

**DOI:** 10.3389/finsc.2026.1684424

**Published:** 2026-02-25

**Authors:** Yamikani Kaliwo, Ellen Kumchenga, Yolice L. B Tembo, Trust Kasambala Donga, Vernon H. Kabambe, Philip C. Stevenson, Steven R. Belmain

**Affiliations:** 1 Department of Crop and Soil Sciences, Lilongwe University of Agriculture and Natural Resources, Lilongwe, Malawi; 2 Department of Trait and Diversity Function, Royal Botanic Gardens, Kew, Richmond, United Kingdom; 3 Natural Resources Institute, University of Greenwich, Gillingham, United Kingdom

**Keywords:** *Aphis fabae*, common bean, conservation biological control, flowering field margin, hoverflies

## Abstract

Bean aphids are a major constraint to bean production worldwide and are commonly managed through intensive pesticide use. In many farming systems, particularly where crop production is increasingly intensified, reliance on chemical control has reduced the use and the appreciation of agroecological pest management strategies, including conservation biological control. The widespread application of pesticides not only suppresses natural enemies of aphids but also contributes to broader declines in insect diversity, especially when combined with the loss of non-crop habitats that support beneficial insects. To address this, we assessed whether engineering bean crop habitats with flowering plants could enhance adult hoverfly populations and increase hoverfly larval abundance within bean fields. The potential of four flowering plant species (*Galinsoga parviflora, Ocimum basilicum, Bidens pilosa* and *Ageratum conyzoides*) grown around bean crops to attract hoverflies was evaluated. Bean crops surrounded by flowering plant species were able to reduce aphid numbers and damage by 51% compared to the control treatment which had no field margin. Bean fields surrounded by *O. basilicum* had the lowest aphid damage score (1.2) and highest bean yield (917 kg/ha). Overall, bean crops surrounded by flowering plant species yielded between 621 to 917 kg/ha, which was 22-42% higher than the untreated control (509 kg/ha). Such evidence may help support policies that promote agroecological practices instead of synthetic pesticides.

## Introduction

Common bean (*Phaseolus vulgaris*: Fabaceae) is a globally important crop that is ideal for smallholder farming systems due to its capacity to fix nitrogen, short duration, compatibility for intercropping, local marketability, and provision of protein and essential vitamins and minerals to resource-poor families. In Africa, common bean is consumed and traded by more than 100 million households. Although average global common bean yields are 2–4 ton/ha, smallholders in Africa typically achieve yields below 0.7 ton/ha owing to a combination of factors including insect pests and diseases (1, 2). For example, black bean aphids, *Aphis fabae* (Hemiptera: Aphididae) is a major insect pest of legumes worldwide, causing yield losses through direct feeding and by transmitting plants viruses (3, 4). This pest is commonly controlled through intensive chemical control, which negatively affects human health and non-target insects (5, 6). The human health impacts of pesticides on smallholders and consumers are increasingly recognized as problematic, exacerbated by mis-use, over-use and incorrect use of chemicals including prohibited products (7–10). This has led to declines in insect species diversity which has been exacerbated by the removal of non-crop habitats that support natural pest regulating species (11–13).

Improved knowledge and understanding of agroecological approaches are critical for smallholders to sustainably intensify crop production. In particular, strategies such as conservation biological control, which harnesses natural enemies like predators and parasitoids to suppress pest populations, can reduce reliance on synthetic pesticides, protect beneficial insects and enhance overall ecosystem services (14, 15). Building smallholder capacity to identify natural enemies, manage field margins and implement habitat engineering can therefore play a key role in improving pest regulation, crop yields and long-term food security. Among the natural enemies promoted through conservation biological control, hoverflies (Diptera: Syrphidae) are particularly important. The Syrphidae comprises almost 6,000 species of hoverfly worldwide and is one of the largest families of Diptera (16). Hoverflies are ecologically diverse, with larvae exhibiting generalist feeding habits, notably aphidophagy and saprophagy (17). These insects provide a dual role in food production as pollinators and predators (18, 19). Despite their ecological importance, the diversity of hoverfly species is poorly documented in Africa potentially hindering effective conservation biocontrol strategies (16, 20). Adult hoverflies are generalist pollinators, requiring nectar and pollen for their survival and thus are attracted to areas with an abundance of flowers to forage from while their larvae are carnivorous and feed on aphids (18).

Despite the promising potential of flowering field margins for enhancing natural pest regulation, adoption among smallholder farmers may be limited by low awareness of beneficial insects. Several studies have shown that many farmers perceive most insects as pests and do not distinguish natural enemies such as hoverflies and pollinators from crop damaging insects (21, 22). In addition, farmers often lack information on the ecological benefits of non-crop flowering plants in supporting natural enemies, resulting in the removal of such plants as weeds (23). Thus studies that provide practical evidence on engineered field margins can be used by extension services, development programs and farmer field schools to demonstrate the role of flowering field margins in enhancing biological control. Integrating these results into participatory training, on-farm demonstration and advisory services could facilitate knowledge and promote wider adoption of agroecological farming practices.

Several flowering plant species have been successfully used in other cropping systems to enhance natural enemy populations, particularly hoverflies, which are key aphid predators. For example, sweet alyssum (*Alyssum maritimum* [L.]) has been used in lettuce systems to attract aphidophagous hoverflies and improve biological control of aphids (24). Similarly, coriander (*Coriandrum sativum* L.), Phacelia (*Phacelia thanacetifolia* Benth), and buckwheat (*Fagopyrum esculentum* Moench) have been used in broccoli and citrus systems (25, 26). Phacelia has been shown to significantly enhance hoverfly fecundity and oviposition in laboratory studies (27). These findings suggest that some flowering species can play a crucial role in supporting beneficial insects.

However, such flower-mediated conservation biological control strategies have not been widely tested in legume systems like common bean. As is the case in more intensive crop production, smallholder farming systems often do not have any field margins with widespread land conversion across marginal land (28). Thus, flower resources are limited across the landscape until the crops, themselves, begin to flower (29), and this could reduce the abundance of hoverflies in these farming landscapes and their potential to contribute to pest management. This study specifically assessed whether locally available flowering plants basil (*Ocimum basilicum* [Lamiales: Lamiaceae]), quickweed (*Galinsoga parviflora* [Asterales: Asteraceae]*)*, black-jack (*Bidens pilosa* [Asterales: Asteraceae]), and goatweed (*Ageratum conyzoides* [Asterales: Asteraceae]) planted around bean fields could enhance adult hoverfly visitation, increase hoverfly larvae abundance, and improve common bean yield across seasons. All four species are annuals, similar to the common bean, ensuring overlapping phenology during the cropping season.

## Materials and methods

### Study sites

**Figure 1:**
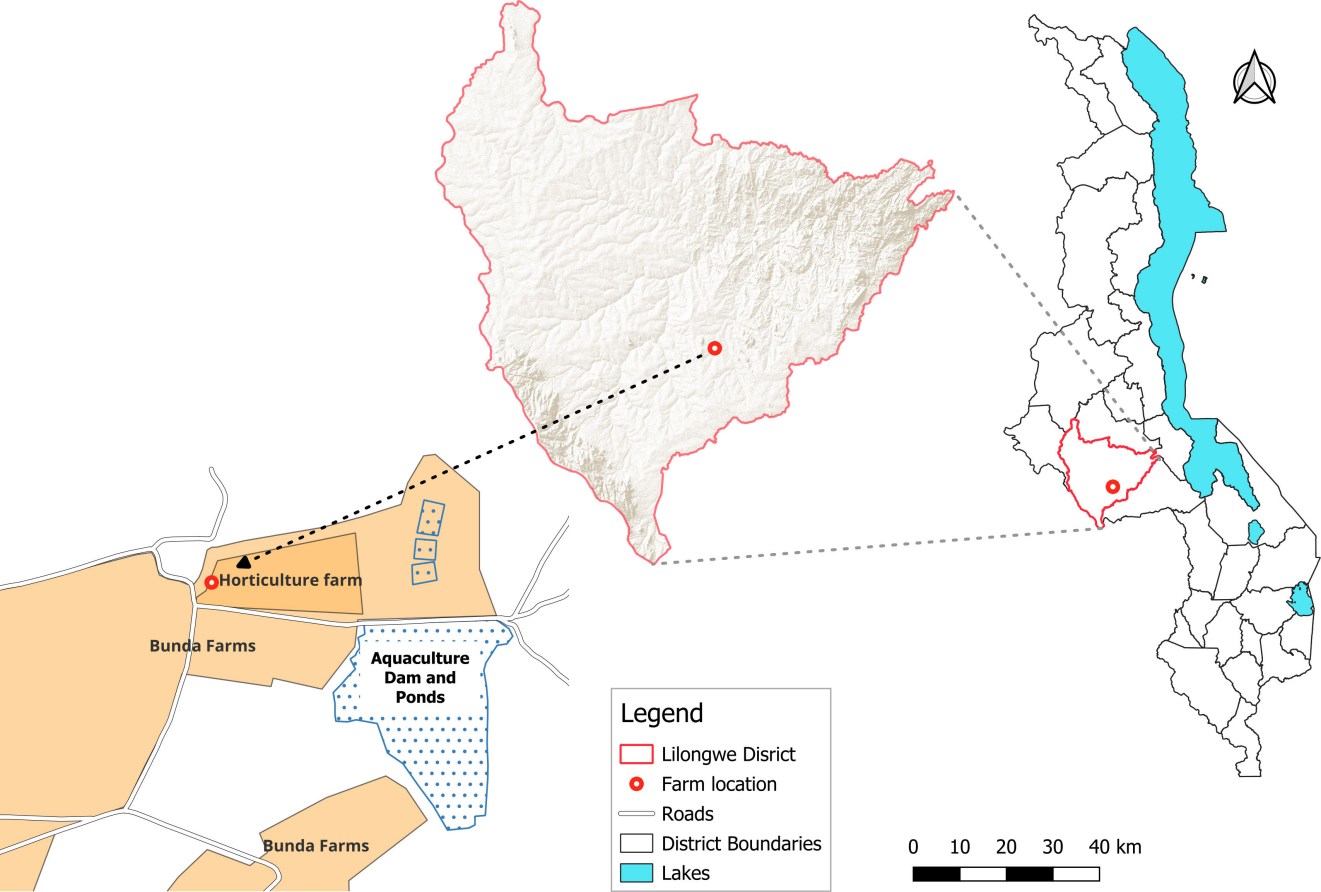
Experimental site location at Bunda Farm based at Lilongwe University of Agriculture and Natural Resources.

The trial was conducted at Bunda campus farm of Lilongwe University of Agriculture and Natural Resources (14°17’S, 33°80’E, altitude of 1054 meters above sea level) (**Figure 1**). The area has a mean annual rainfall of over 700 mm, a mean maximum temperature of 29°C, and a mean minimum temperature of 17°C. Field trials were conducted during the cool dry season using farrow irrigation (June to September 2021) and the rainy season (January to April 2022). Daily minimum and maximum temperatures and rainfall were recorded throughout the study period using on site weather station at Bunda campus farm. Temperature was measured using a digital maximum – minimum thermometer, while rainfall was recorded using a calibrated manual rain gauge. These data were used to examine the effect of climatic variables on aphid abundance.

### Experimentally planted floral field margins

We evaluated the attractiveness of four flowering plant species (*Bidens pilosa*, *Ageratum conyzoides*, *Galinsoga parviflora*, and *Ocimum basilicum)* to hoverflies for aphid biological control. These species were selected for evaluation based on their ecological suitability, local relevance and floral traits associated with hoverfly attraction including morphological and phenological traits such as color, open corollas, accessible nectar and prolonged flowering periods (**Table 1**), where such traits are known to attract hoverflies (30, 31). These plant species also offer multifunctional value, e.g. *O. basilicum* is a culinary and medicinal herb (32), *B. pilosa* and *G. parviflora* are consumed as local vegetables (33) and *A. conyzoides* is used for traditional medicinal purposes (34).

**Table 1:** Characteristics of flowering plant species used in field margin treatments.

Species name	Common name	Flower colour	Corolla depth	Flowering period
*Bidens pilosa*	Blackjack	White/Yellow	Shallow	November–April
*Ageratum conyzoides*	Goatweed	Blue/Purple	Shallow	September-March
*Galinsoga parviflora*	Quickweed	White/Yellow	Very shallow	August - January
*Ocimum basilicum*	Sweet basil	White	Moderate	September- March

**Figure 2:**
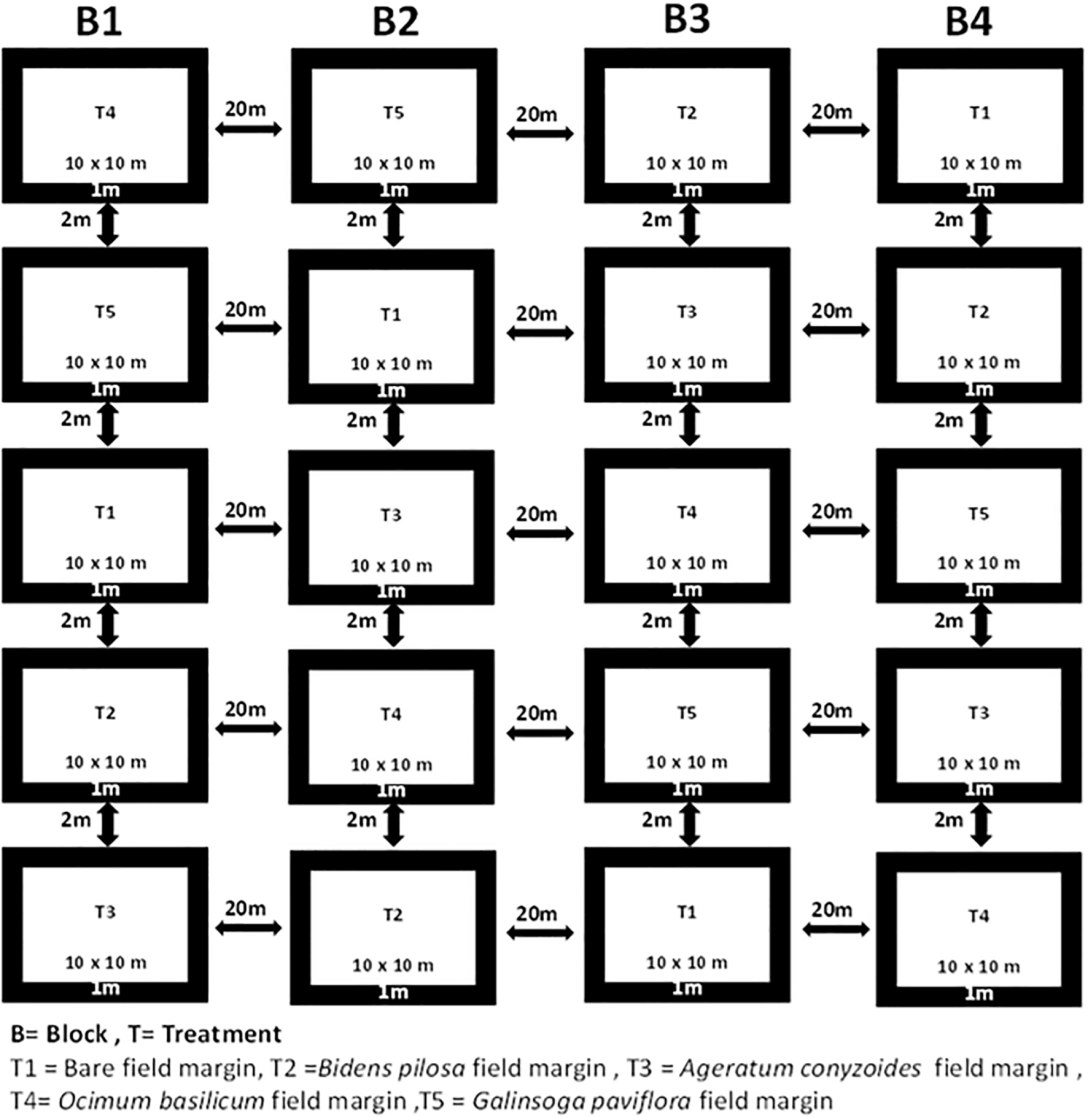
Experimental field plot layout at Bunda farm.

Seedlings of these species were collected from fields in and around Bunda campus farm, raised in nursery for two weeks and subsequently transplanted into the field margins of the experimental plot. The experiment was laid out in a randomized complete block design (RCBD) with a total of five treatments: four bean plots surrounded by different field margin flowering plant species and one control plot with bare margins. Treatments were replicated four times where the blocks were spaced 20 m apart to prevent interaction between the replicates. Thus, with each block of five plots, there was a total of twenty plots. Experimental plots measuring 10 m x 10 m were prepared and were spaced 2 m apart in each block. The field margins were 1 m wide (**Figure 2**).

The flowering plant species seedlings were planted and spaced 20 cm apart and established two weeks before the planting of beans to ensure their maturity and that they would be starting to flower before the beans. Field observation showed that *B. pilosa* began flowering 10–14 days after transplanting and continued for approximately ten weeks, fully overlapping with the bean flowering stages. *A. conyzoides and G. parviflora* initiated flowering about two weeks after bean planting and continued for about four to five weeks, overlapping with mid-flowering stages of beans. *O. basilicum* started flowering two weeks after planting and continued flowering for approximately eight to ten weeks. These flowering durations were recorded under field conditions and presented as approximate flowering spans (**Table 1**).

Two seeds of bean variety NUA 45 were planted in double rows spaced 60 cm x 30 cm. The bean seed was obtained from Crop and Soil Sciences student research farm at Lilongwe University of Agriculture and Natural Resources. No synthetic pesticides were applied in the experimental plots. The field was hand hoe tilled. Manual weeding was carried out twice, once at three weeks after emergence and once during flowering, and no fertilizers were applied in any of the plots.

### Aphid and hoverfly data collection

Aphid data were collected by visually examining ten randomly selected bean plants in each plot. The percent incidence of aphids was calculated as the proportion of the ten sampled plants that were infested with black bean aphids. Aphid abundance and severity were also assessed on the same ten plants by visually estimating the number of aphids and the extent of damage at different growth stages (seedling, vegetative, and reproductive). Aphid abundance was recorded on a scale of 0–5 where: 0 = no aphid infestation, 1 = a few scattered aphids (1−100), 2 = a few isolated colonies (101−300), 3 = several isolated colonies (301−600), 4 = large isolated colonies (601−1000) and 5 = large continuous colonies (>1001). The aphid severity was scored using a 4-point scale according to where: 0 = no infestation or damage, 1 = light damage and infestation (< 25%) of plant parts, 2 = average damage and infestation (26−50%) of plant parts, 3 = high infestation and damage (51−75%) of plant parts showing yellowing of lower leaves and 4 = severe infestation damage (>75%) of plants with yellow and severely curled leaves or a dead plant (35, 36). Thereafter, severity and abundance scores for the ten plants were averaged for each plot. The same plants were also used to count the number of hoverfly larvae and averaged for each plot.

The population of adult hoverflies was estimated three times, once at each bean growth stage (seedling, vegetative, and reproductive), using sweep nets and pan traps. Sweep netting was conducted in the morning hours from 8:00 am to mid-day, spending 20 minutes per plot. When adult hoverflies were captured, the nets were closed with the insects transferred to collecting jars containing cotton wool soaked in ethyl acetate, which was used to kill the hoverflies. Camel-hair brushes subsequently used to remove the specimens from the jars and the hoverflies were placed into 60 ml bottles containing 70% ethanol (37). Adult hoverflies were also collected using a set of three pan traps (yellow, blue and white; 20 cm diameter x 5 cm height, ARKY Plastic Industry Ltd., Malawi) mounted on small stands within each bean plot (38–40). The pan traps were filled to three-quarters with water, with a few drops of liquid detergent added to break surface tension (41, 42). One set of pan trap was placed in the middle of the bean plot and another set was placed at the field margin. Traps were left in the field for 48 hours, after which captured hoverflies were collected and preserved in 70% ethanol. All specimens collected using sweep nets and pan traps were taken to the laboratory for identification to species level using several identification keys (43–48).

### Hoverfly diversity and biodiversity indices

Following hoverfly species identification, species abundance data were compiled for each field margin flowering plant species treatment by pooling counts across sampling methods within each treatment and season. Hoverfly diversity was quantified using the Shannon-Wiener diversity index (H’), which incorporates both species richness (number of species) and evenness (relative abundance of individual among species). The index is calculated as:


$$H^{\prime }=\;-\;\sum_{i=1}^{s}p_{i}\;\text{In}\left(p_{i}\right)$$

where *i_th_* is the proportion of individuals to the *i*^th^ species and s is the total number of species (49). Species evenness was measured to describe the distribution of hoverfly individuals among species, species richness was recorded as the total number of hoverfly species per treatment, and species abundance was defined as the total number of individuals per species.

### Bean crop yield assessment

Within each bean plot, a central area measuring 3m × 4m was used to collect bean pods at crop maturity. The harvested pods were then processed, dried, and weighed in the laboratory, and the resulting yield was converted to kilograms per hectare.

### Data analysis

A generalized linear mixed model (GLMM) was used to examine the effects of field margin flowering plant species and season as fixed effects and their interaction on aphid incidence, aphid abundance, aphid severity and adult and larval hoverfly count, with blocks and plots included as random effects to account for replication. Appropriate distributions and link functions were applied for each response variable: binomial with logit link for aphid incidence, cumulative logit for aphid abundance and severity, which were recorded on ordinal scale data, and a negative binomial with log link for adult and larval hoverfly counts. Aphid abundance in relation to climatic variables was further analyzed using a GLMM with a negative binomial distribution, with temperature and rainfall included as explanatory variables. Bean yield was analyzed using a linear-mixed effect model with field margin flowering plants species and season as fixed effects and block and plots as random effects. Spearman correlation analysis was used to assess the relationship between hoverfly larval abundance and aphid abundance. The Shannon-Weiner and Simpson’s reciprocal indices were analyzed to compare hoverfly diversity among field margin treatments. All the statistical analyses were performed using Genstat 21 Edition (VSN International Ltd).

## Results

### Impact of treatment and season on aphids

**Figure 3:**
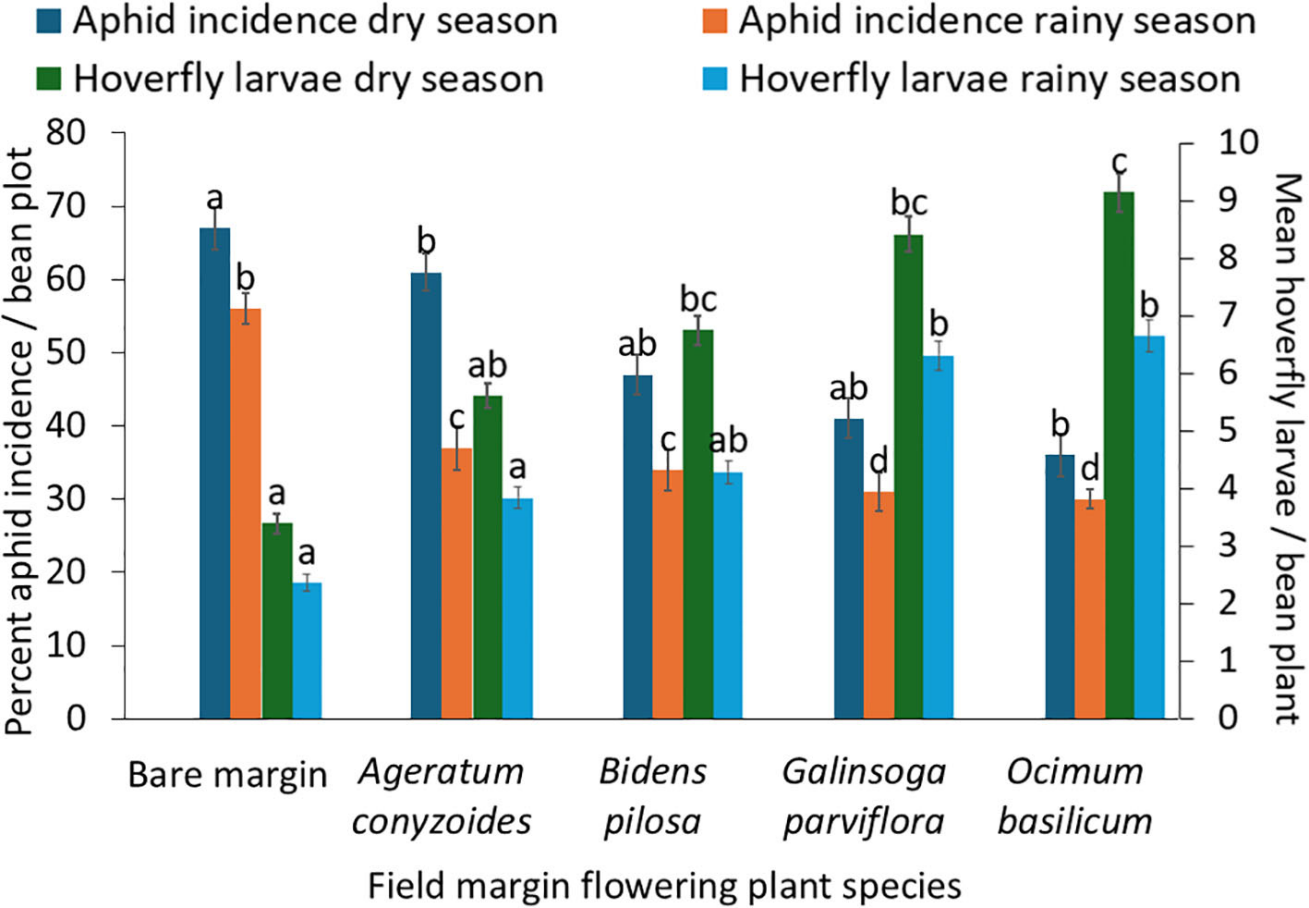
Effects of field margin flowering plant species and growing season on aphid incidence and hoverfly larvae abundance (different letters at top of the bars indicate significant difference).

**Figure 4:**
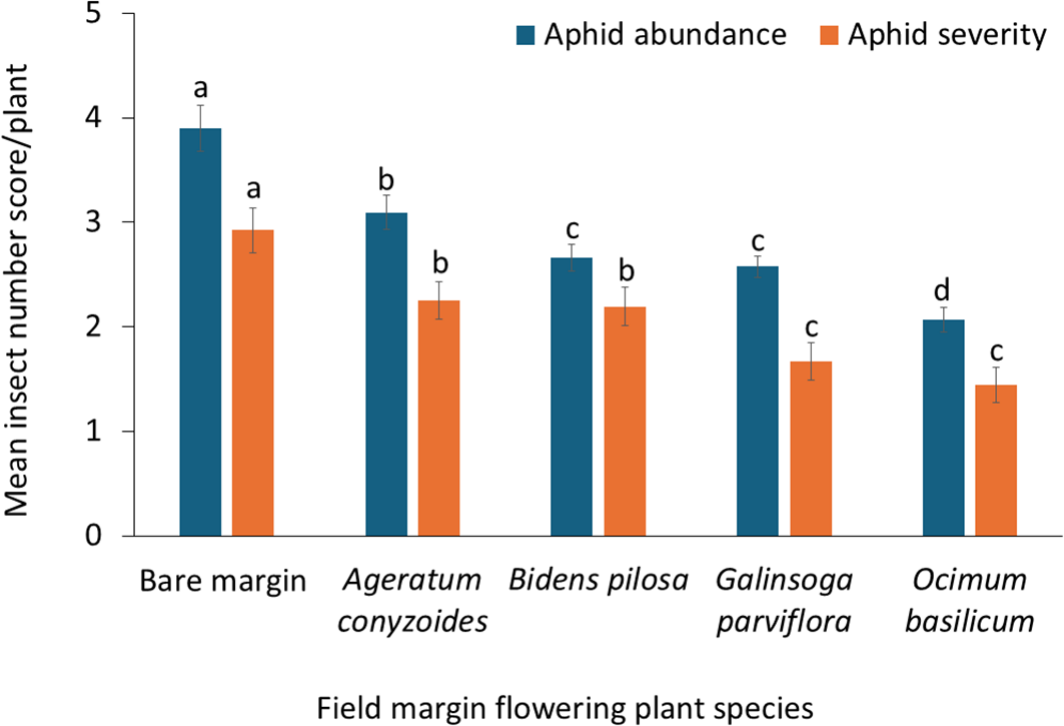
Effect of different field margin flowering plants on aphid abundance and severity in bean plots. Aphid abundance was assessed on an ordinal scale from 0 to 5 (*0 = no aphids; 1 = 1–100 aphids; 2 = 101–300; 3 = 301–600; 4 = 601–1000; 5 = >1000 aphids*), while aphid severity was scored on a 0–4 scale (*0 = no damage; 4 = severe damage affecting >75% of plant parts*).(Bars represent mean ± SE. Different letters above bars indicate significant differences).

The analysis revealed significant effects of both field margin plant species F = 5.39, df =4,190, *P* = 0.001 and season F = 8.21, df =1,190, *p* = 0.001, on aphid incidence (**Table 2**). Across both seasons, aphid incidence was highest in bean plots with bare margins, with 67% aphid incidence in the cool dry season and 56% in the rainy season. In contrast, plots surrounded by *O. basilicum* recorded the lowest aphid incidence, 36% in the cool dry season and 30% in the rainy season (**Figure 3**). There were also significant differences in aphid abundance F = 5.27, df = 4,190, *P =* 0.001 and severity F = 4.71, df = 4,190, *p* = 0.001 among treatments with different field margin flowering plant species (**Table 2**). Aphid abundance and severity were lowest in bean plots surrounded by *O. basilicum*, whereas bean fields with bare margin exhibited the highest aphid severity (**Figure 4**).

**Table 2:** Statistical evaluation of the attractiveness of different flowering plants on hoverfly populations, impact of cropping season, impact of pan traps and sweep nets on adult hoverfly capture rates, the effect of pan trap positioning on hoverfly capture rates for the parameters of aphid score, hoverfly larvae, adult hoverflies, and bean crop yields when bean crops were surrounded by different flowering plant species in the field margins.

Field margin plant species	Df	Aphid incidence	Aphid score	Aphid severity	Hoverfly larvae	Adult hoverfly	Bean yield
F	5	5.39	5.27	4.71	13.22	12.65	3.209
R^2^		0.1456	0.1333	0.11731	0.32161	0.210	0.398
Pr > F		<0.001	<0.001	<0.001	<0.001	<0.001	0.026
Season F	1	8.21	5.32	3.33	18.26	5.63	1.663
Pr > F		<0.005	0.084	0.070	<0.001	0.018	0.207
FMP*season F	3	0.40	0.67	0.68	0.64	0.43	1.342
Pr > F		<0.0806	0.980	0.610	0.636	0.790	0.277
Trapping method							
F	1					11.84	
R^2^						0.210	
Pr > F						< 0.0001	
Pan trap positioning							
F	1					29.18	
R^2^						0.200	
Pr > F						< 0.0001	
Hoverfly species							
F	1					33.30	
R^2^						0.929	
Pr > F						< 0.0001	

### Influence of field margin plants on hoverfly larvae

The results showed that both field margin flowering plants species F = 13.22, df = 4,190, *p* = 0.001 and season F = 18.26, df = 1,190, *p =* 0.001,had significant effects on hoverfly larvae abundance in bean plots (**Table 2**). The highest abundance of hoverfly larvae was recorded in plots surrounded by *O. basilicum*, with an average of nine larvae per bean plant during the cool dry season and seven hoverfly larvae per bean plant during the rainy season. In contrast, bean plots with bare margins had the lowest abundance, with only three larvae per bean plant in the cool dry season and two larvae per bean plant during the rainy season (**Figure 3**). A moderate and statistically significant negative relation between hoverfly larval abundance and aphid abundance was observed, r = - 0.324, p< 0.001 (**Table 3**). Furthermore, rainfall had a significant negative effect on aphid abundance *p* < 0.031, with higher rainfall associated with lower aphid numbers (**Table 4**).

**Table 3:** Pearson correlation value between the population of hoverfly larvae and aphid abundance.

	Aphid abundance	Hoverfly larvae
Aphid abundance	1	
Hoverfly larvae	-0.324^**^	1

** value is significant at *p* = 0.01.

**Table 4:** Effect of rainfall and temperature on aphid abundance.

Aphid Abundance	Coef.	SE	P-value
Temperature (^0^C)	0.003	0.005	0.529
Rainfall (mm)	-0.38	0.02	0.031
Constant	1.855	.429	0

### Factors affecting adult hoverflies

**Figure 5:**
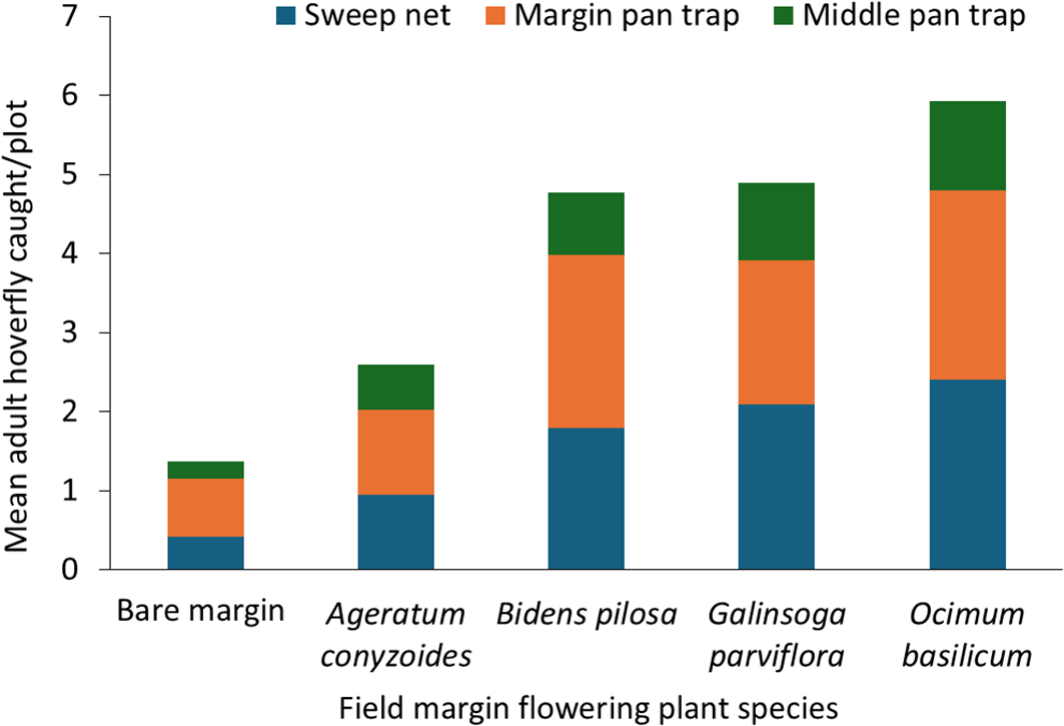
Effects of treatment, trapping method, and trap positioning on adult hoverfly abundance.

Field margin flowering plant species had a significant effect on the number of adult hoverflies recorded in bean plots F = 12.65, df = 4,377, *p* = 0.001, and growing season also had a significant effect F = 5.63, df = 1,377, *p* = 0.018 (**Table 1**). The highest number of adult hoverflies was captured in bean plots surrounded by *O. basilicum* during the cool dry season, followed by those surrounded by *G. parviflora*, while the lowest number were recorded in bean plots with bare margins (**Figure 5**). The method used to capture hoverflies significantly influenced the total number recorded F = df = 1,137, 11.8, *p =* 0.001(**Table 1**). Pan traps captured significantly more adult hoverflies than sweep nets across all the treatments (**Figure 5**). Additionally, the position of the pan traps had a significant effect on hoverfly abundance F = 29.18, df = 1,388, *p* = 0.001 (**Table 1**). Pan traps placed in the field margins caught more adult hoverflies than those placed in the center of the plots across all the treatments (**Figure 5**).

### Hoverfly species diversity, abundance and bean yield

**Figure 6:**
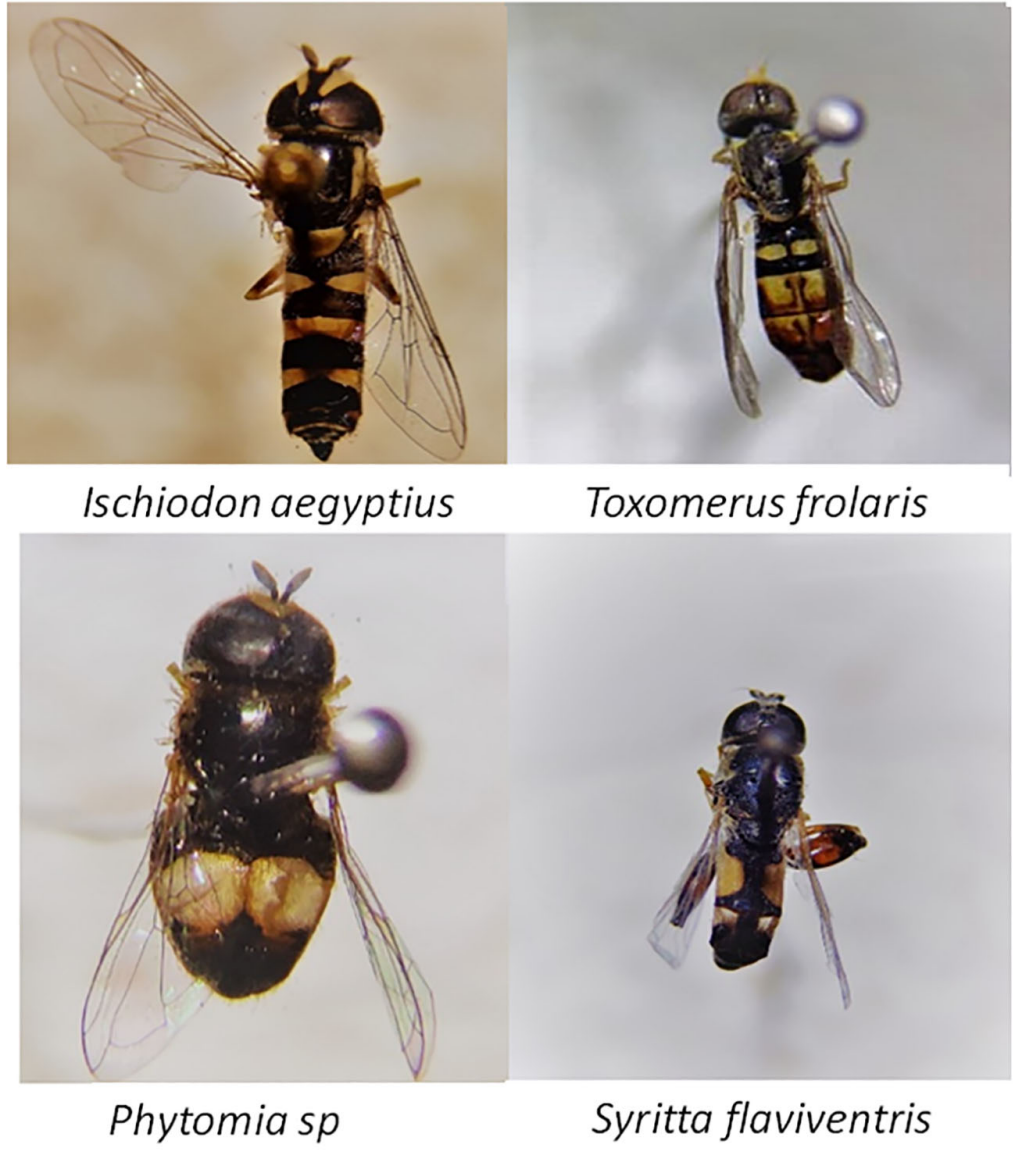
Photos of the hoverfly species captured across the different treatment plots.

**Figure 7:**
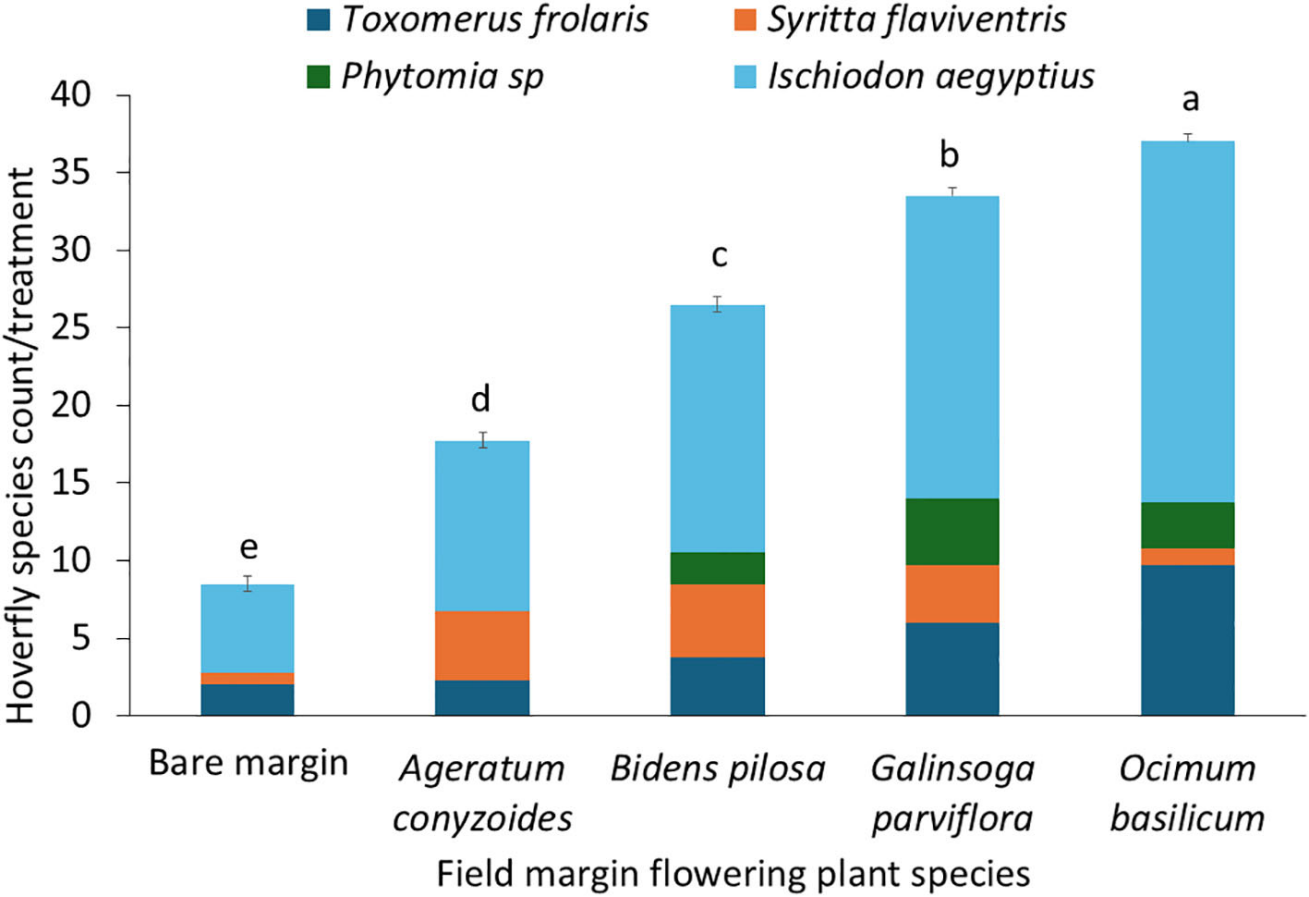
Hoverfly species abundance across experimental bean field plots.

**Figure 8:**
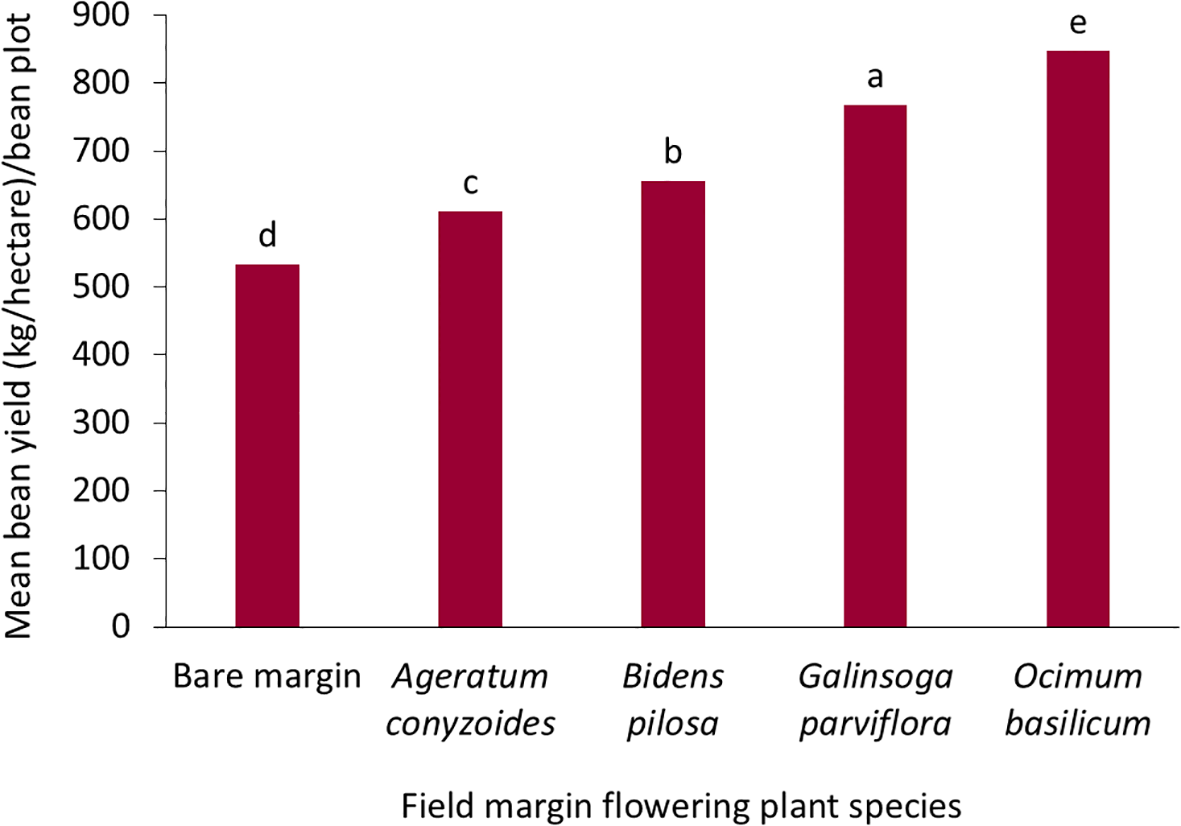
Effect of the field margins with flowering plants species on bean yield (different letters at top of the bars indicate significant differences).

Hoverfly species diversity and abundance varied significantly across the treatments F = 33.0, df = 4,79, *p* = 0.001 (**Table 1**). A total of 493 hoverflies were recorded (**Table 5**), comprising four species (Diptera: Syrphidae): *Ischiodon aegyptius, Syritta flaviventris, Toxomerus floralis and Phytomia* sp. (**Figure 6**). Of these *I. aegyptius* and *T. floralis* are aphidophagous species whose larvae prey on aphids, whiles *S. flaviventris* and *Phytomia* sp. are saprophagous species that develop in decaying organic matter*. O. basilicum* supported the highest abundance and diversity of adult hoverflies, followed by *G. parviflora*, while bare margins consistently had the lowest abundance and diversity (**Figure 7**). *I. aegyptius* was the most frequently caught species overall, followed by *T. floralis*, with *Phytomia* sp. being the least common (**Figure 7**). Bean yield also differed significantly among the treatments F 4,39 = 3.209, *p =* 0.026 (**Table 1**). The highest yields were obtained from bean plots surrounded by *O. basilicum*, whereas the lowest yields were observed in bean plots with bare margins (**Figure 8**).

**Table 5:** Hoverfly species diversity indices by field margin plant species.

Field margin species	Shannon-Weiner H	Simpson (1/D)	Species richness
*Ageratum conyzoides*	4.15	61.27	71
Bare margin	3.41	29.89	34
*Bidens pilosa*	4.50	83.98	106
*Galinsoga parviflora*	4.73	105.13	134
*Ocimum basilicum*	4.87	124.77	148
Total			493

## Discussion

The presence of *O. basilicum* in field margins maintained a low aphid population and supported higher hoverfly abundance throughout the study and had a beneficial impact on common bean yield. These findings align with other studies in different contexts showing flowering plants can improve crop yields by facilitating conservation biological control (50). Natural enemies, including hoverflies, are often attracted to plants having white, yellow, and blue flowers (51), and studies have shown these colors elicit hoverfly visiting behavior on plants (19, 52). Flower structure with a short collar depth is a preferred trait for beneficial insects like hoverflies as these flowers provide easier access to pollen and nectar (53). *Ocimum basilicum* flowers possess these traits, which together with serial flower production over several weeks, may explain higher hoverfly abundance. Based on these results, encouraging the cultivation of *O. basilicum* in field margins could enable smallholder farmers to increase their crop yields through natural pest regulation without resorting to the use of synthetic pesticides to control their aphid pest problems. Our study focused on flowering border species known to possess traits attractive to hoverflies. Thus, further studies could include non-flowering borders or plant species that are not attractive to hoverflies, providing useful contrasts in evaluating hoverfly preferences.

Aphid incidence, abundance and severity were higher in bean crops grown during the cool dry season compared to the rainy season. Heavy and prolonged rainfall has been reported to create unfavorable conditions for aphid growth and reproduction, partly by washing off aphids off host plants (54, 55). Hoverfly populations were also higher during the cool dry bean growing season compared to the rainy season. With more aphid prey available, adult female hoverflies will lay more eggs close to aphid populations, leading to more hoverfly larvae and higher hoverfly populations (56, 57). Similar trends on hoverfly oviposition have been observed under both field conditions and in greenhouses (58).

In the current study, bean plots surrounded by different flowering plants in their field margins resulted in lower aphid numbers compared to plots with no flowering plants. We argue that higher numbers of adult hoverflies attracted to nectar resources likely contributed to increased hoverfly oviposition and subsequent preying on aphids. We acknowledge that other flower visiting predators and parasitoids may also play a role reducing aphid numbers (59). Other research also shows that planting the right flowers in field margins can increase hoverfly numbers in crop fields (23, 60–62). Although the relationship between aphid abundance and hoverfly larvae may also be influenced by other ecological factors such as microclimatic variation or the activity of other predators, the observed negative correlation between hoverfly larval abundance and aphid abundance supports that higher hoverfly numbers likely contributed to aphid suppression.

In this study, adult hoverflies were more frequently captured in pan traps placed along field margins than in in the center of bean plots, indicating hoverfly activity in these areas. Other aphid predators were occasionally observed in the fields, such as ladybird beetles and lacewings but their abundance was not systematically recorded. This pattern aligns with previous studies showing that adult hoverflies concentrate in field margins after oviposition to access nectar resources and resting sites, while their larvae develop on aphid-infested crops (23, 63). Among the hoverfly species collected, *I. aegyptius* was the most common species found across all the bean plots with different flower margins. This species is widespread and stays active for much of the year (64). Because it has a short life cycle and multiple generations each year, it can effectively prey on many types of aphids in different cropping seasons (65). Although the pan traps used in this study were not hoverfly specific and captured a wide range of other insect taxa, only hoverflies were identified and quantified in the analysis. Consequently, other flower visiting or predatory species that may have contributed to aphid suppression were not assessed. Future work could include broader insect community sampling to better separate the relative contribution of different natural enemy guilds to aphid regulation.

Furthermore, while our design minimized potential interference between treatments, the relatively short distance between treatment plots within the block may have allowed some movement of mobile insects such as alate aphids and hoverfly adults across plots (18). The distance that hoverflies will travel can be several hundred meters or even many hundreds of kilometers for those which migrate (66, 67). To minimize potential effects of insect movement among the treatments, the experiment was arranged in a randomized complete block design, with spatial variability accounted for through replication and appropriate statistical controls.

## Conclusions

Flowering field margins increased the abundance of adult hoverflies and hoverfly larvae in bean plots, which was associated with reduced aphid numbers and increased bean yields. Although hoverflies were the focal natural enemy evaluated, we acknowledge that flowering borders may also support other predators and parasitoids that could contribute to aphid suppression. The most influential field margin plant in this study was *O. basilicum*, which supported the highest hoverfly presence and lowest aphid population. Our findings demonstrate that habitat engineering through strategically selected flowering plants can enhance natural enemy populations and potentially improve yield outcomes in smallholder bean production systems. Further studies integrating direct predation measurements and broader natural enemy community assessments would help clarify the proportional contribution of hoverflies relative to other natural enemies.

## Data Availability

The raw data supporting the conclusions of this article will be made available by the authors, without undue reservation.

## References

[1] Abate T Ampofo JKO . Insect pests of beans in Africa: their ecology and management. Annu Rev Entomol. (1996) 41:45–73. doi: 10.1146/annurev.en.41.010196.000401, PMID: 15012324

[2] de Bon H Huat J Parrot L Sinzogan A Martin T Malézieux E . Pesticide risks from fruit and vegetable pest management by small farmers in sub-Saharan Africa. A review. Agron Sustain Dev. (2014) 34:723–36. doi: 10.1007/s13593-014-0216-7, PMID: 41721156

[3] Wamonje FO Donnelly R Tungadi TD Murphy AM Pate AE Woodcock C . Different plant viruses induce changes in feeding behavior of specialist and generalist aphids on common bean that are likely to enhance virus transmission. Front Plant Sci. (2020) 10:1811. doi: 10.3389/fpls.2019.01811, PMID: 32082355 PMC7005137

[4] Skovgård H Stoddard FL . Reproductive potential of the black bean aphid Aphis fabae Scop.) on a range of faba bean (Vicia faba L.) accessions. Legume Sci. (2023) 5:e199. doi: 10.1002/leg3.199, PMID: 41718500

[5] Belmain SR Tembo Y Mkindi AG Arnold SEJ Stevenson PC . Elements of agroecological pest and disease management. Elementa. (2022) 10:00099. doi: 10.1525/elementa.2021.00099, PMID: 41538050

[6] Ngegba PM Cui G Khalid MZ Zhong G . Use of botanical pesticides in agriculture as an alternative to synthetic pesticides. Agriculture. (2022) 12:600. doi: 10.3390/agriculture12050600, PMID: 41700957

[7] Donald CE Scott RP Blaustein KL Halbleib ML Sarr M Jepson PC . Silicone wristbands detect individuals’ pesticide exposures in West Africa. R Soc Open Sci. (2016) 3:160433. doi: 10.1098/rsos.160433, PMID: 27853621 PMC5108971

[8] Sheahan M Barrett CB Goldvale C . Human health and pesticide use in Sub-Saharan Africa. Agric Econ. (2017) 48:27–41. doi: 10.1111/AGEC.12384, PMID: 41711423

[9] Guy Bertrand P . Uses and misuses of agricultural pesticides in africa: neglected public health threats for workers and population. Pesticides - Use Misuse Their Impact Environ. (2019). doi: 10.5772/intechopen.84566, PMID: 32864128

[10] Fuhrimann S Wan C Blouzard E Veludo A Holtman Z Chetty-Mhlanga S . Pesticide research on environmental and human exposure and risks in sub-saharan africa: A systematic literature review. Int J Environ Res Public Health. (2021) 19:259. doi: 10.3390/ijerph19010259, PMID: 35010520 PMC8750985

[11] Raven PH Wagner DL . Agricultural intensification and climate change are rapidly decreasing insect biodiversity. Proc Natl Acad Sci. (2021) 118:e2002548117. doi: 10.1073/PNAS.2002548117, PMID: 33431564 PMC7812793

[12] Wagner DL Grames EM Forister ML Berenbaum MR Stopak D . Insect decline in the Anthropocene: Death by a thousand cuts. Proc Natl Acad Sci U.S.A. (2021) 118:e2023989118. doi: 10.1073/pnas.2023989118, PMID: 33431573 PMC7812858

[13] El-Shafie HAF . Global Decline of Insects. Abdel Farag El-Shafie H , editor. London, UK: IntechOpen (2022). doi: 10.5772/intechopen.94711, PMID:

[14] Li C Kandel M Anghileri D Oloo F Kambombe O Chibarabada TP . Recent changes in cropland area and productivity indicate unsustainable cropland expansion in Malawi. Environ Res Lett. (2021) 16:084052. doi: 10.1088/1748-9326/AC162A

[15] Nazombe KS Nambazo O Mdolo P Bakolo C Mlewa R . Assessing changes in the ecosystem service value in response to land use and land cover dynamics in Malawi. Environ Monit Assess. (2024) 196:741. doi: 10.1007/s10661-024-12915-5, PMID: 39017942

[16] Rojo S Gilbert F Marcos-García MA Nieto JM Mier MP . A world review of predatory hoverflies (Diptera, Syrphidae: Syrphinae) and their prey. Rojo S Gilbert F Marcos-García MA Nieto JM Mier MP , editors. Alicante: Centro Iberoamericano de la Biodiversidad (CIBIO (2003).

[17] Rotheray G Gilbert F . The natural history of hoverflies. Tresaith, Wales, UK: Forrest Text (2011).

[18] Doyle T Hawkes WLS Massy R Powney GD Menz MHM Wotton KR . Pollination by hoverflies in the anthropocene. Proc R Soc B. (2020) 287:20200508. doi: 10.1098/RSPB.2020.0508, PMID: 32429807 PMC7287354

[19] Rodríguez-Gasol N Alins G Veronesi ER Wratten S . The ecology of predatory hoverflies as ecosystem-service providers in agricultural systems. Biol Control. (2020) 151:104405. doi: 10.1016/J.BIOCONTROL.2020.104405, PMID: 41723091

[20] Azo’o Ela M Bissou Wangbara B Jordaens K . Diversity of flower-visiting hoverflies (Diptera: Syrphidae) on ground cover vegetation from the market-gardening area of Meskine (Far-North Region, Cameroon). Afr J Ecol. (2022) 60:58–66. doi: 10.1111/AJE.12922, PMID: 41711423

[21] Elisante F Ndakidemi PA Arnold SEJ Belmain SR Gurr GM Darbyshire I . Enhancing knowledge among smallholders on pollinators and supporting field margins for sustainable food security. Journal of Rural Studies. (2019) 70:75–86. doi: 10.1016/j.jrurstud.2019.07.004, PMID: 41723091

[22] Mkenda PA Ndakidemi PA Stevenson PC Arnold SEJ Darbyshire I Belmain SR . Knowledge gaps among smallholder farmers hinder adoption of conservation biological control. Biocontrol Science and Technology. (2020) 30:256–277. doi: 10.1080/09583157.2019.1707169, PMID: 41669619

[23] Ndakidemi BJ Mbega ER Ndakidemi PA Belmain SR Arnold SEJ Woolley VC . Field margin plants support natural enemies in sub-saharan africa smallholder common bean farming systems. Plants. (2022) 11:898. doi: 10.3390/plants11070898, PMID: 35406877 PMC9002875

[24] Gillespie M Wratten S Sedcole R Colfer R . Manipulating floral resources dispersion for hoverflies (Diptera: Syrphidae) in a California lettuce agro-ecosystem. Biol Control. (2011) 59:215–20. doi: 10.1016/J.BIOCONTROL.2011.07.010, PMID: 41723091

[25] Colley MR Luna JM . Relative attractiveness of potential beneficial insectary plants to aphidophagous hoverflies (Diptera: syrphidae). Environ Entomol. (2000) 29:1054–9. doi: 10.1603/0046-225X-29.5.1054, PMID: 38536611

[26] Irvin NA Pierce C Hoddle MS . Evaluating the potential of flowering plants for enhancing predatory hoverflies (Syrphidae) for biological control of Diaphorina citri (Liviidae) in California. Biol Control. (2021) 157:104574. doi: 10.1016/J.BIOCONTROL.2021.104574, PMID: 41723091

[27] Laubertie EA Wratten SD Hemptinne JL . The contribution of potential beneficial insectary plant species to adult hoverfly (Diptera: Syrphidae) fitness. Biol Control. (2012) 61:1–6. doi: 10.1016/J.BIOCONTROL.2011.12.010, PMID: 41723091

[28] Peter BG Messina JP Snapp SS . A multiscalar approach to mapping marginal agricultural land: smallholder agriculture in Malawi. Ann Am Assoc Geogr. (2018) 108:989–1005. doi: 10.1080/24694452.2017.1403877, PMID: 41669619

[29] Bouwman TI Andersson JA Giller KE . Adapting yet not adopting? Conservation agriculture in Central Malawi. Agric Ecosyst Environ. (2021) 307:107224. doi: 10.1016/J.AGEE.2020.107224, PMID: 41723091

[30] Klecka J Hadrava J Biella P Akter A . Flower visitation by hoverflies (Diptera: Syrphidae) in a temperate plant-pollinator network. PeerJ. (2018) 6:e6025. doi: 10.7717/peerj.6025, PMID: 30533311 PMC6282941

[31] Rweyemamu EW Kabota S Tryphone GM De Meyer M Mwatawala MW . Floral visitation of European honey bees and hoverflies in selected cultivated cucurbitaceous crops in Morogoro, Eastern-Central Tanzania. PloS One. (2025) 20:e0322219. doi: 10.1371/journal.pone.0322219, PMID: 40354430 PMC12068622

[32] Dhama K Sharun K Gugjoo MB Tiwari R Alagawany M Iqbal Yatoo M . A comprehensive review on chemical profile and pharmacological activities of *ocimum basilicum*. Food Rev Int. (2023) 39:119–47. doi: 10.1080/87559129.2021.1900230, PMID: 41669619

[33] Lesten ECC Kingsley GM . Proximate and phytochemical composition of selected indigenous leafy vegetables consumed in Malawi. Afr J Food Sci. (2020) 14:265–73. doi: 10.5897/AJFS2020.1979, PMID: 38147025

[34] Yadav N Ganie SA Singh B Chhillar AK Yadav SS . Phytochemical constituents and ethnopharmacological properties of Ageratum conyzoides L. Phytotherapy Res. (2019) 33:2163–78. doi: 10.1002/ptr.6405, PMID: 31290201

[35] Mkenda P Mwanauta R Stevenson PC Ndakidemi P Mtei K Belmain SR . Extracts from field margin weeds provide economically viable and environmentally benign pest control compared to synthetic pesticides. PloS One. (2015) 10:e0143530. doi: 10.1371/journal.pone.0143530, PMID: 26599609 PMC4658159

[36] Ochieng LO Ogendo JO Bett PK Nyaanga JG Cheruiyot EK Mulwa RMS . Field margins and botanical insecticides enhance Lablab purpureus yield by reducing aphid pests and supporting natural enemies. J Appl Entomology. (2022) 146:838–49. doi: 10.1111/jen.13023, PMID: 36249719 PMC9545213

[37] Pineda A Marcos-García M. Á . Use of selected flowering plants in greenhouses to enhance aphidophagous hoverfly populations (Diptera: Syrphidae). Annales la Société entomologique France (N.S.). (2008) 44:487–92. doi: 10.1080/00379271.2008.10697584, PMID: 41669619

[38] Irvin NA Wratten SD Frampton CM Bowie MH Evans AM Moar NT . The phenology and pollen feeding of three hover fly (Diptera: Syrphidae) species in Canterbury, New Zealand. N Z J Zool. (1999) 26:105–15. doi: 10.1080/03014223.1999.9518182, PMID: 41669619

[39] Sutherland JP Sullivan MS Poppy GM . The influence of floral character on the foraging behaviour of the hoverfly, *Episyrphus balteatus*. Entomol Exp Appl. (1999) 93:157–64. doi: 10.1046/j.1570-7458.1999.00574.x, PMID: 41717205

[40] Janković M Miličić M Nedeljković Z Milovac Ž Ačanski J Vujić A . Diversity and structure of hoverfly (Diptera: syrphidae) communities in agricultural areas in vojvodina province (Serbia) A case study on brassica napus L. J Entomol Res Soc. (2019) 21:129–44. Available online at: https://www.entomol.org/journal/index.php/JERS/article/view/1406 (Accessed February 20, 2026).

[41] Obanyi JN Ogendo JO Mulwa RMS Nyaanga JG Cheruiyot EK Bett PK . Field margins and cropping system influence diversity and abundance of aphid natural enemies in Lablab purpureus. J Appl Entomology. (2023) 147:439–451. doi: 10.1111/jen.13125, PMID: 41711423

[42] Karimi JM Nyaanga JG Mulwa RMS Ogendo JO Bett PK Cheruiyot EK . Lablab (Lablab purpureus L.) genotypes and field margin vegetation influence bean aphids and their natural enemies. Front Insect Sci. (2024) 4:1328235. doi: 10.3389/finsc.2024.1328235, PMID: 39006941 PMC11240140

[43] Thompson FC Vockeroth JR . Family syrphidae. In: Evenhuis NL , editor. Catalog of the diptera of the australasian and oceanian regions. Bishop Museum Press and E. J. Brill, Honolulu (1989). p. 437–58.

[44] Perez-Banon C Marcos-Garcia MA . Description of the immature stages of Syritta flaviventris (Diptera: Syrphidae) and new data about the life history of European species of Syritta on Opuntia maxima. Eur J Entomol. (2000) 97:131–6. doi: 10.14411/eje.2000.022

[45] Lyneborg L Barkemeyer W . The genus syritta: A world revision of the genus syritta le peletier & Servilla 1828(Diptera: syrphidae). Stenstrup (Denmark: Apollo Books (2005).

[46] Ramage T Sylvain C Mengual X . Flower flies (Diptera, Syrphidae) of French Polynesia, with the description of two new species. Eur J Taxon. (2018) 2018:1–37. doi: 10.5852/ejt.2018.448

[47] van Steenis J Nedeljković Z Tot T van der Ent L-J van Eck A Mazánek L . New records of hoverflies (Diptera: Syrphidae) and the rediscovery of Primocerioides regale Violovitsh for the fauna of Serbia. Biol Serbica. (2019) 41. doi: 10.5281/zenodo.3526446, PMID: 41712760

[48] De Meyer M Goergen G Jordaens K . Taxonomic revision of the afrotropical phytomia guérin-méneville (Diptera: syrphidae). Zootaxa. (2020) 4803:201-250–201–250. doi: 10.11646/zootaxa.4803.2.1, PMID: 33056016

[49] Magurran AE . Measuring biological diversity. Oxford, UK: Wiley-Blackwell (2013). p. 272.

[50] Tschumi M Albrecht M Collatz J Dubsky V Entling MH Najar-Rodriguez AJ . Tailored flower strips promote natural enemy biodiversity and pest control in potato crops. J Appl Ecol. (2016) 53:1169–76. doi: 10.1111/1365-2664.12653, PMID: 41711423

[51] Hannah L Dyer AG Garcia JE Dorin A Burd M . Psychophysics of the hoverfly: categorical or continuous color discrimination? Curr Zool. (2019) 65:483–92. doi: 10.1093/CZ/ZOZ008, PMID: 31413720 PMC6688577

[52] Day RL Hickman JM Sprague RI Wratten SD . Predatory hoverflies increase oviposition in response to colour stimuli offering no reward: Implications for biological control. Basic Appl Ecol. (2015) 16:544–52. doi: 10.1016/J.BAAE.2015.05.004, PMID: 41723091

[53] Branquart E Hemptinne JL . Selectivity in the exploitation of floral resources by hoverflies (Diptera: Syrphinae). Ecography. (2000) 23:732–42. doi: 10.1111/J.1600-0587.2000.TB00316.X, PMID: 41711423

[54] Forchibe EE Fening KO Narh-Madey B Afreh-Nuamah K Cobblah MA Wamonje FO . Differential effects of weather, plant phenology and predators on the seasonal variation of aphids on cabbage. J Appl Entomology. (2023) 147:261–70. doi: 10.1111/jen.13106, PMID: 38601126 PMC11005107

[55] Almogdad M Lavrukaitė K Semaškienė R . Temporal Analysis of the Relationship between Black Bean Aphid (Aphis fabae) Infestation and Meteorological Conditions in Faba Bean (Vicia faba). Agronomy. (2024) 14:1182. doi: 10.3390/AGRONOMY14061182, PMID: 41700957

[56] Almohamad R Verheggen FJ Francis F Haubruge E . Predatory hoverflies select their oviposition site according to aphid host plant and aphid species. Entomol Exp Appl. (2007) 125:13–21. doi: 10.1111/J.1570-7458.2007.00596.X, PMID: 41711423

[57] Gonzalez N Fournier M Buitenhuis R Lucas E . Evaluating a new aphid biocontrol agent: The role of aphid density in modulating oviposition behaviour in the American hoverfly, *Eupeodes americanus*, and the aphid midge, *Aphidoletes aphidimyza*. J Appl Entomology. (2024) 148:5–12. doi: 10.1111/jen.13202, PMID: 41711423

[58] Rashid MS Yaqoob M Panda A Fatimah N Ayoub L . Harnessing the potential of syrphids: their role as bio-agents and pollinators. Int J Plant Soil Sci. (2024) 36:8–22. doi: 10.9734/IJPSS/2024/V36I115117, PMID: 41723892

[59] Obanyi JN Ogendo JO Mulwa RMS Nyaanga JG Cheruiyot EK Bett PK . Flowering margins support natural enemies between cropping seasons. Front Agron. (2024) 6:1277062. doi: 10.3389/fagro.2024.1277062, PMID: 41717471

[60] Marshall EJP West TM . Impacts of field margins, landscape and crop on the distributions of Syrphidae on an arable farm. Asp Appl Biol. (2007) 81:91–9. Available online at: https://www.cabidigitallibrary.org/doi/full/10.5555/20073239727 (Accessed February 20, 2026).

[61] Hogg BN Nelson EH Mills NJ Daane KM . Floral resources enhance aphid suppression by a hoverfly. Entomol Exp Appl. (2011) 141:138–44. doi: 10.1111/J.1570-7458.2011.01174.X, PMID: 41711423

[62] Blaix C Moonen A-C . The influence of field margin characteristics on syrphid abundance. Arthropod Plant Interact. (2023) 17:31–42. doi: 10.1007/s11829-022-09934-9, PMID: 41721156

[63] O’Connor RS Kunin WE Garratt MPD Potts SG Roy HE Andrews C . Monitoring insect pollinators and flower visitation: The effectiveness and feasibility of different survey methods. Methods Ecol Evol. (2019) 10:2129–40. doi: 10.1111/2041-210X.13292, PMID: 41711423

[64] Kirk-Springgs AH Sinclair BJ . Manual of afrotropical diptera vol. 3. Pretoria: South African National Biodiversity Institute (SANBI) Publishing (2021).

[65] Petrović A Vujić M . The northernmost record of *Ischiodon aEgyptius* (Wiedemann 1830) (Diptera, Syrphidae, Syrphinae) with possible evidence of its reproduction in Europe. Entomologia Croatica. (2024) 23:44–51. doi: 10.17971/ec.23.1.7

[66] Menz MHM Brown BV Wotton KR . Quantification of migrant hoverfly movements (Diptera: Syrphidae) on the West Coast of North America. R Soc Open Sci. (2019) 6:190153. doi: 10.1098/rsos.190153, PMID: 31183151 PMC6502382

[67] Rader R Cunningham SA Howlett BG Inouye DW . Non-bee insects as visitors and pollinators of crops: Biology, ecology, and management. Annu Rev Entomol. (2020) 65:391–407. doi: 10.1146/ANNUREV-ENTO-011019-025055/1, PMID: 31610136

